# A Rare Case of Isolated Ureteral Diaphragmatic Herniation: Case Report and Review of Literature

**DOI:** 10.1155/2019/1092018

**Published:** 2019-12-02

**Authors:** Nassib Abou Heidar, Hussein Elsemesmani, Ahmad Elamine, Mustafa Natout

**Affiliations:** ^1^Department of Surgery, Division of Urology, American University of Beirut Medical Center, Beirut, Lebanon; ^2^Department of Radiology, American University of Beirut Medical Center, Beirut, Lebanon

## Abstract

Diaphragmatic ureteral hernias are rare causes of ipsilateral renal obstruction. Management strategies varies from conservative to ureteral stenting to operative herniorraphy and reduction. We present a case of a 71-year-old man who was found to have an incidental right ureteral diaphragmatic herniation causing an asymptomatic kidney obstruction. He was managed conservatively with no evidence of increased hydronephrosis on serial imaging and no deterioration of biochemical renal function. We review all similar cases published in the literature and discuss the optimal treatment strategies.

## 1. Introduction

It is a rarity to encounter ureteral herniation with less than 150 reported in the literature. Common hernia locations are inguinal, femoral, and sciatic locations among others [1]. However, the rarest location for a ureteral herniation is through a defect in the diaphragmatic muscle [2]. Previously, only 12 cases have reported in the literature with varying presentations and management strategies; management varied from conservative to ureteral stenting or surgical repair. On the other hand, the clinical presentation varied from incidental radiographic finding to obstructive pyelonephritis and urosepsis. We hereby present the case of a ureteral diaphragmatic hernia that was incidentally discovered on computed tomography (CT).

## 2. Case Presentation

**Figure 1:**
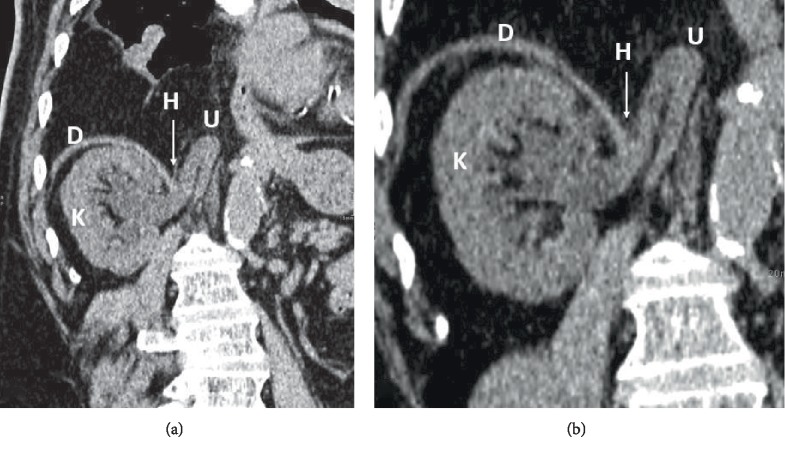
Reformatted oblique coronal images of nonenhanced CT scan of the abdomen show the right ureter herniating through a medial diaphragmatic defect causing its obstruction and secondary mild to moderate hydronephrosis. Renal cortical thickness is still preserved. D: right hemidiaphragm. K: right kidney. U: ureter. H: diaphragmatic hernia.

**Figure 2:**
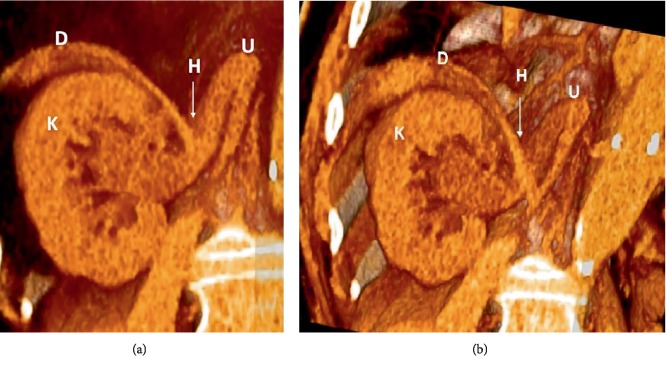
3-D reconstructed images showing the right ureter herniation and the secondary hydronephrosis. D: right hemidiaphragm. K: right kidney. U: ureter. H: diaphragmatic hernia.

**Figure 3:**
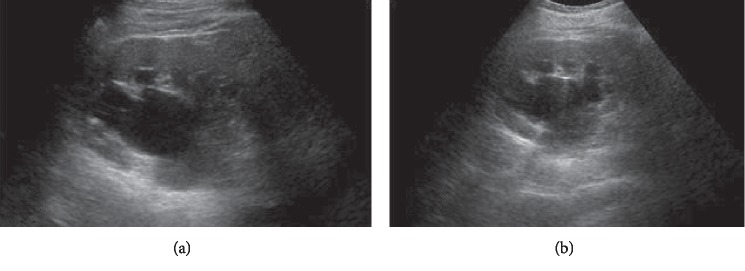
Serial ultrasound images of the right kidney done at 6 month intervals after diagnosis of the kidney obstruction. Showing similar ureteral and pelvicalyceal dilation (a and b) over the span of 6 months with no change in the renal cortex thickness.

A 71-year-old male, with a medical history relevant for uncontrolled hypertension, diabetes mellitus type 2, and chronic kidney disease (baseline creatinine = 1.8 mg/dl), presented to the emergency department with abdominal pain, vomiting and loose stools. The patient denied any new-onset lower tract urinary symptoms, hematuria, fever, or flank pain. His laboratory tests were notable for leukocytosis of 12,000/cu.mm, serum creatinine of 1.9 mg/dl, and negative urinalysis. A CT scan done to rule out an intraabdominal process did not reveal any intraperitoneal pathology; however, a rightsided ureteral diaphragmatic herniation was seen, associated with mild-moderate hydronephrosis and significant blunting of calyceal fornices (Figures 1 and 2). The patient was counselled about his management options which included stenting of the ureter to relieve the obstruction or active surveillance of the hydronephrosis for which he opted. Serial ultrasounds performed every 6 months showed no progression of the hydronephrosis and no renal cortical thinning (Figure 3). Follow-up on serum creatinine showed no change in his baseline levels and was in the 1.8–2 mg/dl range for over 18 months.

## 3. Discussion

Ureteral hernias are rare entities, with less than 150 cases previously reported. The rarest ureteral herniation reported is thoracic ureteral herniation through a muscular diaphragmatic defect. It is speculated that these types of ureteral herniations are rare because the herniation is occurring through a nondependent space [2]. It is currently unknown if these herniations are acquired or congenital with late presentation.

A retrospective review of reported cases was done and papers were retrieved from PubMed, Medline, and Scopus. 12 cases were found that had different presentations and management plans ranging from conservative management to ureteral stenting or surgical repair depending on the presentation and the clinical scenario. These were summarized in Table 1.

In the literature, some ureteral herniations are detected incidentally while others presented with symptoms attributable to the ureteral herniation/kidney obstruction. Symptomatic patients presented with flank pain, gross hematuria, renal dysfunction, nephrolithiasis and urosepsis. Among the thoracic ureteral herniations reported in the literature, five presented with right flank pain [2, 3, 5, 6], three were incidental findings in the workup for azotemia [7], contralateral flank pain [8], and PET scan for the workup of a lung nodule [9]. Two cases presented as part of the workup for renal [10] and ureteral [11] stones, one presented as right upper quadrant abdominal pain [12], and one presented as septic obstructive pyelonephritis [13].

Of these reported cases, one was managed conservatively [7], three underwent surgical repair [3, 6, 10], and ureteral stenting [2–4, 11–13]. Among those that underwent stenting, one necessitated a nephrostomy access due to failure of retrograde stenting [13], another was followed by surgical repair [2], and one underwent intra-renal surgery with stenting [11]. Two cases did not report management [8, 9]. Most cases occurred in the elderly, with the mean age of diagnosis being 76, and out of the 12 reported cases, 9 were female.

Congenital Bochdalek hernias are a result of failure of fusion of the foramina of the diaphragm. They are usually asymptomatic with prevalence rate reaching 10% [14]. In about 90% of the cases symptoms of Bochdaleck, hernias are left-sided [13]. However, in all reported cases of ureteral herniation, the herniation lateralized to the right-side. This is counterintuitive since the right kidney is higher in location and the liver is right-sided and would occupy a larger space. The authors have no explanation for this observation; however, this observation seems to support the embryologic origin of this diaphragmatic defect. On the other hand, the fact that this seems to be a presentation in the geriatric population discourages the embryologic origin of ureteral herniation.

We report the 13th case of thoracic ureteral herniation to our knowledge, a rather uncommon finding. It further supports the literature in that thoracic ureteral herniations generally occur on the right side. In our case, the herniation was successfully managed conservatively with serial ultrasounds and serum creatinine in order to follow up on the hydronephrosis and renal function, respectively, both done at 6-month-intervals with the former showing no progression of the hydronephrosis and no cortical thinning, and the latter showing no elevations over the period of follow up. This further supports that uncomplicated ureteral herniations can be managed conservatively with surveillance.

Management strategies depend on the clinical context. In cases of obstruction and resultant pain or pyelonephritis, then drainage is imperative. A trial of retrograde ureteral stenting is reasonable and minimally invasive [12] with notable success in the reported cases; however, Hatzidagis et al. reported on failure of double J ureteral stent insertion owing that to the length and tortuosity of the ureter [13]. More importantly, this case encourages that a trial of surveillance of renal function and obstruction is a valid method at management.

## 4. Conclusion

Ureteral herniations are generally an uncommon entity, with thoracic ureteral herniations being even more exceedingly rare in the literature. Imaging is required to detect, confirm, and follow the herniation for progression. Follow up can take the form of serial ultrasounds and serum creatinine while the patient is managed conservatively. Our case suggests that uncomplicated thoracic ureteral herniations can be managed conservatively without intervention, particularly in patients not amenable to surgery.

## Figures and Tables

**Table 1:** Review of ureteral diaphragmatic herniation.

Authors (Year of publication)	Age/gender	Laterality	Presentation	Management
Swithinbank (1958)	60/F	Right	Right flank pain	Surgical repair
Paterson and Lupton (1989)	75/M	Right	Right flank pain	Surgical repair
Chawla and Mond (1994)	56/M	Right	Incidental on workup for contralateral flank pain	Not mentioned
Catalano et al. (1998)	63/F	Right	Upon workup for renal stones	Surgical repair of the defect
Sukumar et al. (2010)	75/F	Right	Incidental finding on workup for azotemia	Conservative
Balakrishnan and Neerhut (2011)	83/F	Right	Right flank pain	Retrograde ureteral stenting
Song et al. (2011)	75/M	Right	Right upper quadrant pain	Retrograde ureteral stenting
Hatzidakis et al. (2014)	86/F	Right	Septic obstructive pyelonephritis	Nephrostomy drainage followed by ureteral stenting
Almeida et al. (2015)	82/F	Right	Incidental on PET scan for workup of lung nodule	Not mentioned
Dru and Josephson (2016)	94/F	Right	Right flank pain	Retrograde ureteral stenting
Lin et al. (2017)	81/F	Right	Right flank pain	Retrograde ureteral stenting followed by surgical repair
Beland et al. (2019)	84/F	Right	Obstructing ureteral stone	Retrograde intra-renal surgery for stone fragmentation and stenting
Current case	71/M	Right	Incidental	Conservative
